# The Effect of Maternal Thyroid Disorders (Hypothyroidism and Hyperthyroidism) During Pregnancy and Lactation on Skin Development in Wistar Rat Newborns

**Published:** 2013-05

**Authors:** Maryam Amerion, Somayye Tahajjodi, Zahra Hushmand, Nasser Mahdavi Shahri, Mohammad Reza Nikravesh, Mahdi Jalali

**Affiliations:** 1 Azad University of Shahrud, Medical Sciences School, Shahrud, Iran; 2 Ferdowsi University of Mashhad, Faculty of Basic Sciences, Mashhad, Iran; 3 Mashhad University of Medical Sciences school, Mashhad, Iran

**Keywords:** Hypothyroidism, Lactation, Laminin, Maternal, Pregnancy, Skin development

## Abstract

***Objective(s):*** Previous studies have shown that thyroid hormones are necessary for normal development of many organs and because of the importance of skin as the largest and the most important organ in human body protection in spite of external environment, the study of thyroid hormones effects on skin development is considerable. In this survey we have tried to study the effects of maternal hypothyroidism on skin development in fetus during pregnancy and lactation by immunohistochemistry technique.

***Materials and Methods:*** Rats were divided into 4 groups, hypothyroids, hyperthyroids, hypothyroids are treated with levothyroxin and a control group. The rat mothers were exposed to PTU with 50 mg/lit dosage and levothyroxin with 1 mg/lit dosage and PTU and levothyroxin simultaneously and with the same dosage respectively in hypothyroid, hyperthyroid and treated hypothyroids with levothyroxin groups. After 14 days, blood sample was taken from mothers, and if thyroid hormones level had change well, mating was allowed. After pregnancy and delivery, 1th day dorsal skin (as the sample for pregnancy assay) and 10th day skin (as for lactation assay) was used for immunohystochemical and morphometric studies.

***Results:*** In this study it was observed that maternal hypothyroidism during pregnancy and lactation causes significant increase in laminin expression, in most areas of skin, and maternal hyperthyroidism during pregnancy and lactation causes significant decrease in laminin expression. Also significant decrease was observed in hair follicles number and epidermis thickness in hypothyroidism groups.

***Conclusion: ***This study showed maternal hypothyroidism causes significant decrease in epidermis thickness and hair follicles number and it causes less hair in fetus. Also maternal hypothyroidism causes large changes in laminin expression in different parts of skin. At the same time,maternal hyperthyroidism causes opposite results. In fact, thyroid hormones regulate laminin expression negatively which means increase in thyroid hormone level, decreases laminin expression. So changes in thyroid hormones level can influence skin development significantly.

## Introduction

As we know, thyroid hormones have key role on different systems development, so maternal hypothyroidism during pregnancy decreases thyroid hormones passing from mother to embryo via placental-uterine barrier. This will cause developmental defects on embryos nervous system. But these defects are not restricted to nervous system merely, and affect other organs, too. Thyroid hormones receptor isoforms exist in different areas of skin so they can affect skin considerably (1, 2). In this study, we surveyed the influence of thyroid hormones on skin development. Many different proteins have various roles in dermis and epidermis layers development and laminin is one of them. Extracellular matrix of skin has a key role in connection, growth and distribution of epithelial cells like keratinocytes, gene expression regulation and differentiation (3).

Extracellular matrix components include fibronectin, laminin and collagen, between them Laminin is the most important component of basal membrane and is secreted by fibroblast and epithelial cells (4); it has an important role in cellular connection, adhesion and regeneration process (5). In skin, laminin-5 provides a special substrate for stable connections and adhesion of proliferating and migrating cells (6). 

Laminin-5 has also a key role in dermal-epidermal connection. Considering these important roles of laminin, developmental changes from genetic disorders or hormone secretion or any agent that affects the expression of their coding genes, can impair skin structure. In this study, it is tried that using laminin detection in extracellular matrix of mentioned layers and basement membrane structure; its expression intensity will be considered as a good instrument for studying. Due to laminin intensity and presence in mentioned layers, we decided to use laminin detection in extracellular layer, as a good study method.

At the same time morphometric studies and determination of hair follicles density and skin layers thickness based on obtained parameters from stereology studies, changes (if occurred) were also studied. Since the extracellular matrix and basement membrane structure are the base of tissue formation, different cell lines genesis, the supplier and guarantee for critical activities, in every tissue of body any changes in the structure of these layers, can lead to changes in the structure and function of related tissues. 

In comparison with other aspects of hypothyroidism, little studies have been done about its effects on histogenesis changes of skin layers considering these; carful study of this issue from two perspectives immunohistochemical (laminin molecule detection) and morphometric technique probably would lead to results that have not been reported yet. 

In this study, the effect of maternal hypothyroidism on dermis, epidermis and hypodermis layers of rat newborns was studied. Also using H&E staining and immunohistochemical technique, developmental changes of mentioned layers based on laminin detection in extracellular matrix and basement membrane of experimental and control groups was studied. Parallel to this method, using the morphometric methods based on stereological technique, possible changes of layers thickness were calculated. 

## Materials and Methods

This study is an experimental and basic study and all ethical rules were considered about the rats. 40 virgin wistar female rats were selected randomly in 4 following groups: hypothyroid, hyperthyroid, treated hypothyroid with levothyroxin and control group (n=10 in each group). They were obtained from animal house of Mashhad University of Medical Sciences. The environmental conditions were equal for all (23-25°C, relative humidity 50-55%, 12 hr light-dark cycle, light on at 6.00 am). The mothers in hypothyroid group, hyperthyroid group and treated hypothyroid with levothyroxin group, from 14 days before mating, were exposed respectively to propylthiouracyle (PTU) (made in IRAN, Irandaru company) and with 50 mg dosage, levothyroxin (made in IRAN, Irandaru company) with 1 mg dosage, and both of them simultaneously and with the same dosages. In 14^th^ day, the blood test was taken from mother's ocular sinus and if thyroid hormone level in hypothyroid group reduced and in hyperthyroid group increased and in treated hypothyroid with levothyroxin group didn’t change, mothers would be allowed to mate. T4 test showed T4 level in control rats is 3.5-17 μg/dl and in hypothyroid rats is less than control rats that means 1.5-3.5 μg/d and in treated hypothyroid with levothyroxin rats is similar to control rats. Also TSH level was studied in these groups and showed significant increase in hypothyroid group rather than control group. After delivery, newborns were separated from mothers in 1^th^ day (for pure thyroid disorders assay during pregnancy) and 10^th^ day of borth (for thyroid disorders assay during pregnancy and lactation). Then they were anesthetized with ether and then had a transcardial perfusion with 10 cc of paraformaldehyde 4%. The dorsl skin sample were isolated and fixed for 48 hr at room temperature and then processed with routine histological methods and finally embedded in paraffin. The prepared blocks were cut serially in 5 micron coronal sections. One out of each ten sections, one was selected. After deparaffination and rehydration, the selected sections were placed in Triton X-100 for 10 min and then incubated with polyclonal anti laminin for 2 hr (anti mouse anti rabbit, made by Abcan company). After washing with PBS, they were placed in Di-aminobenzidine for 10 min and again washed for 10 min with PBS. Finally, they were stained with hematoxylin and mounted with enthelan. According to the appearance of the laminin in different parts of skin, sections will show positive coloring reaction to the used anti body, from light to dark brown. Because the rate of coloring reaction is the determinant of laminin density, Gong method is used (7-9) for grading the coloring reaction from zero to 4+ according to the intensity of the reaction (by 4 people for judgment about the intensity of reaction). Photographs were taken of microscopic view and grading method was performed by two separate individuals. 

Also for morphometric studies, stereology method was used. Aafter providing serial sections from each specimen, an 8×8 cm^2^ square was located on each picture and hair follicles number were counted by millimeter ruler in this square and afterwards in real size magnificated pictures (×20) based on the fact that 80 mm in square is equal to 0.021 mm in millimeter ruler, then 80 divided to 0.021, it is observed that distances are 380 times in this magnification then 80 divided to 380 so it is provided real size of squar and and real size of unit. Consequently the changes of hair follicles number in per o.o44 mm^2 ^of the real tissue were calculated and epidermal thickness changes in different groups were investigated by microscopic ruler. Finally Statistical analysis was done by SPSS software version 11.5 using nonparametric Kruskal Wallis and Mann-Withney U tests for laminin reaction. The significance level was defined at α < 0.05. Also f t-test was used or morphometric studies. 

## Results

**Figure 1:**
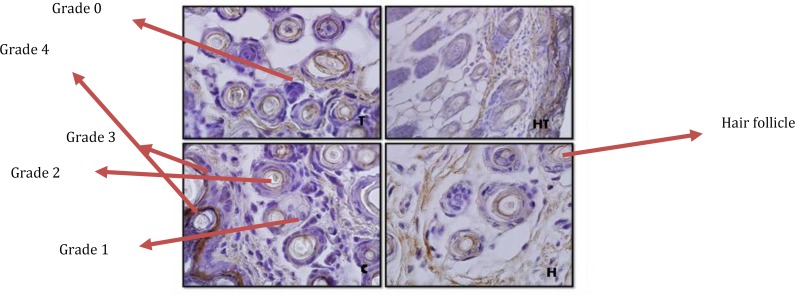
Stained hair follicles by immunohistochemistry technique in 10 days old newborns (magnification 100X). as it is observed in laminin expression in hypothyroid group (H), hyperthyroid (T), treated hypothyroid with levothyroxin (HT) rather than control group, no significant difference was observed. Grading is base on Gong method

In this study, laminin expression in different parts of skin was investigated. The results showed in hair follicles of 10 day old newborns, in all mentioned groups, no significant difference was observed (Figure 1). 

**Figure 2:**
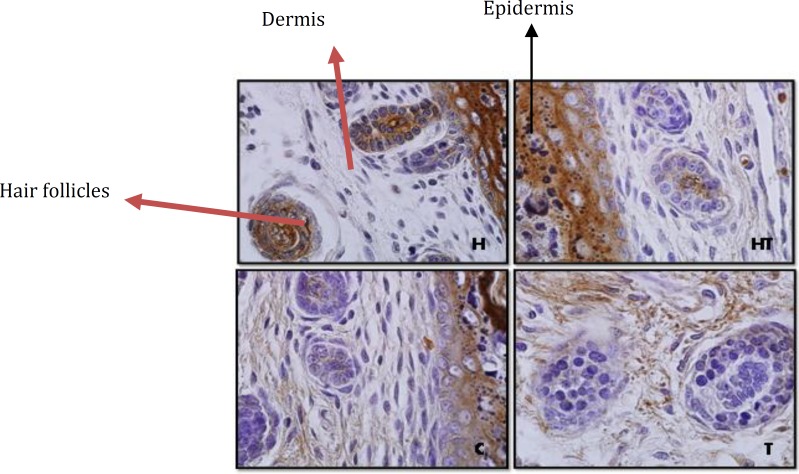
Stained hair follicles by immunohistochemistry technique in 1 day old newborns (magnification 100X). As it is observed in laminin expression in hypothyroid group (H) significant increase and in hyperthyroid (T) significant decrease and in treated hypothyroid with levothyroxin (HT) rather than control group, no significant difference was observed

**Figure 3:**
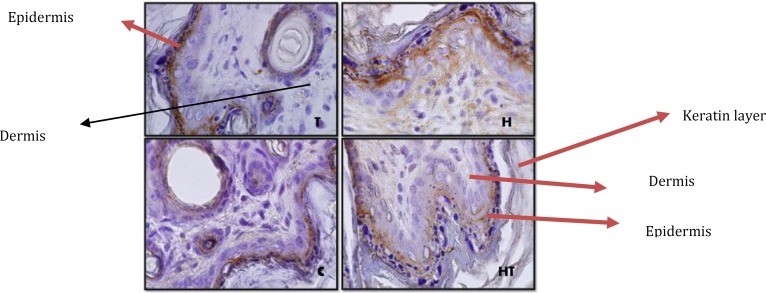
Stained dermis and epidermis by immunohistochemistry technique in 10 days old newborns (magnification 100X). laminin expression in hypothyroid group (H) rather than control group in all layers of epidermis except stratum cornea increase significantly but in hyperthyroid group (T) no significant difference was observed

**Figure 4:**
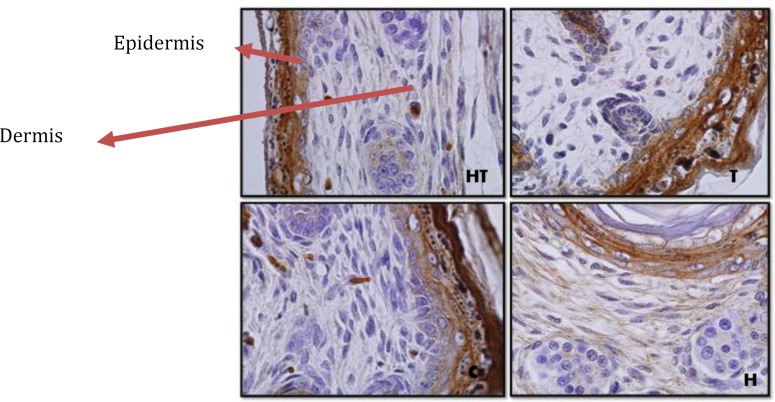
Dermis and epidermis by immunohistochemistry technique in 1 day old newborns (magnification 100X). laminin expression in hyperthyroid group (T) rather than control group in all layers of epidermis except stratum cornea increase significantly but in hypothyroid group (H) no significant difference was observed

**Figure 5:**
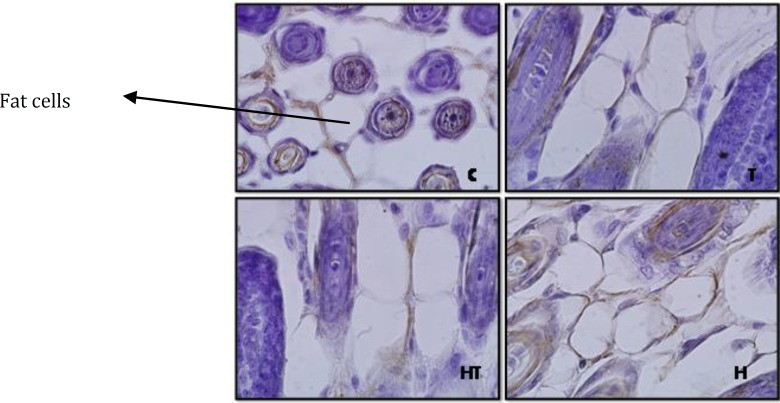
Stained fat cells by immunohistochemistry technique in 10 days old newborns (magnification 100X). As it is observed laminin expression in hypothyroid (H) rather than control group (C) increases significantly and in hyperthyroid group (T) decreases

**Figure 6:**
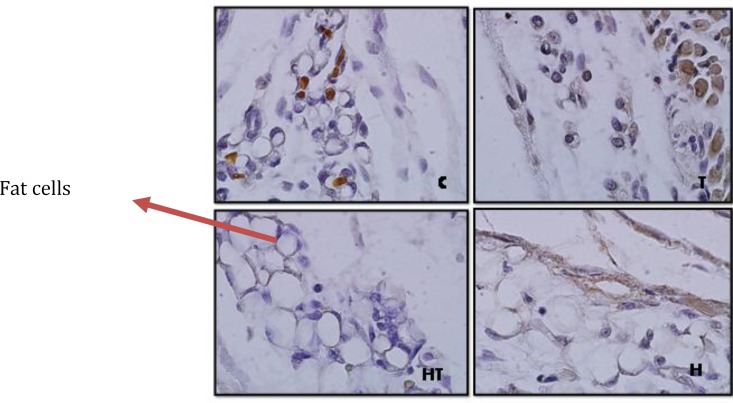
Stained fat cells by immunohistochemistry technique in 1 day old newborns (magnification 100X). As it is observed laminin expression in hypothyroid group (H) rather than control group (C) increases significantly and in hyperthyroid group (T) no significant difference was observed

**Graph 1:**
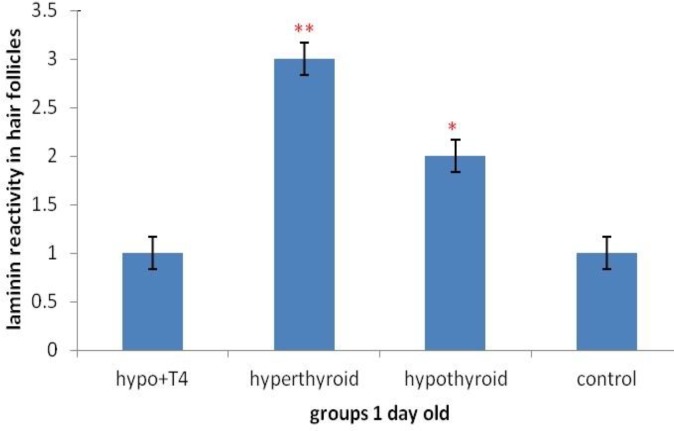
Laminin expression changes in hair follicles in 1 day old newborns (**P<0.01, *P<0.05)(Mean±SD: 3±1.44)

As it is observed laminin expression increases significantly in hypothyroid and hyperthyroid group rather than control group

**Graph 2:**
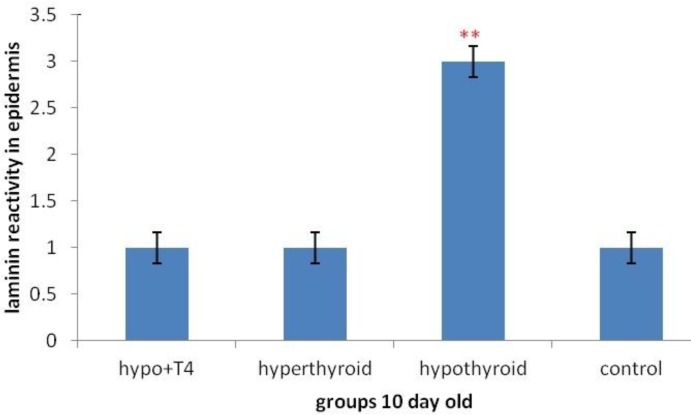
Laminin expression changes in epidermis in 10 days old newborn (**P<0.01) (Mean±SD: 2±1.77)

**Graph 3:**
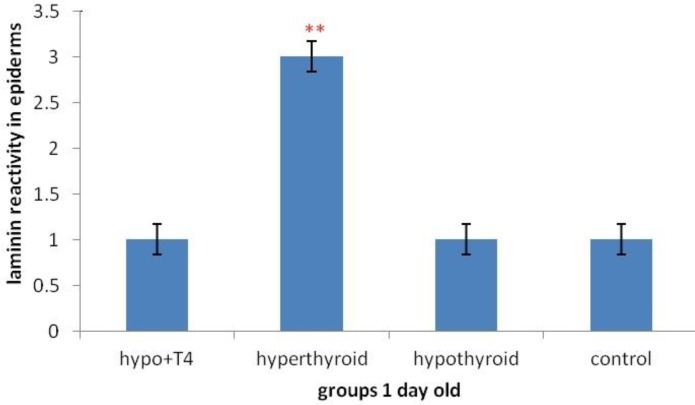
Laminin expression changes in 1 day old newborns epidermis (**P<0.01). (Mean±SD: 2±1.77)

**Graph 4:**
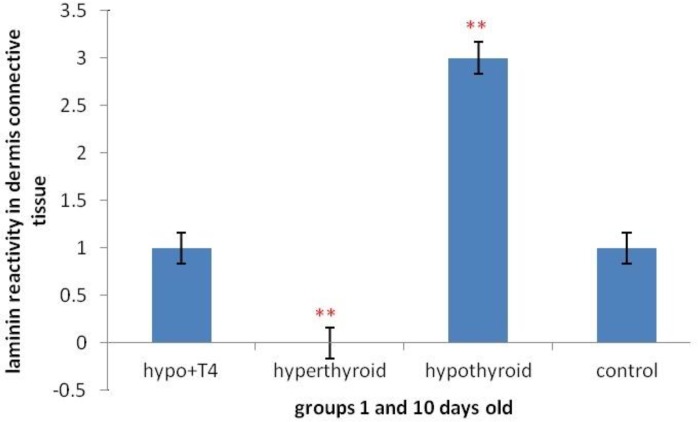
Laminin expression changes in dermis connective tissue of 1 and 10 day old newborns (**P<0.01, *P<0.05) (Mean±SD: 1.5±1.44)

As it is observed laminin expression in hypothyroid group rather than control group increases and in hyperthyroid group decreases significantly

**Graph 5:**
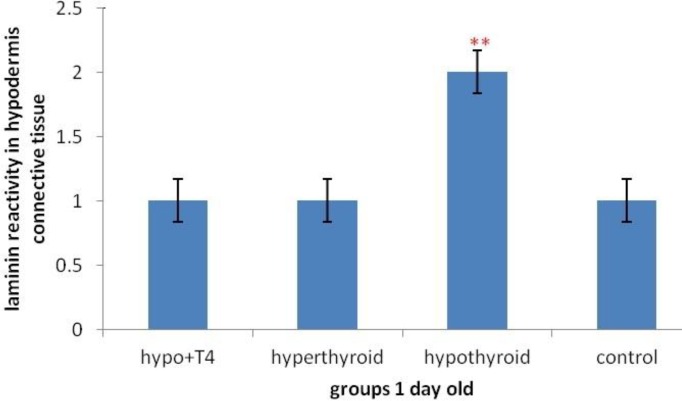
Laminin expression changes in hypodermis connective tissue and fat cells of 10 days old newborns (**P<0.01) (Mean±SD: 2±1.78)

As it is observed laminin expression in hypothyroid group rather than control group increases and in hyperthyroid group decreases significantly

**Graph 6:**
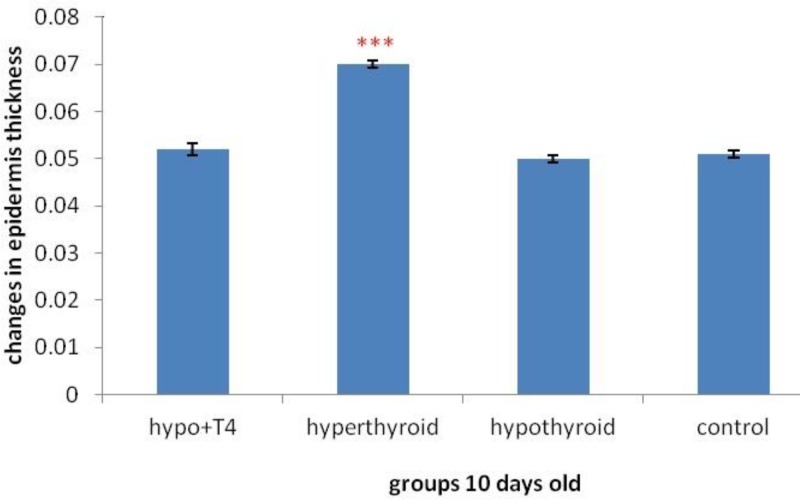
Laminin expression changes in hypodermis connective tissue and fat cells of 1 day old newborns (**P<0.01) (Mean±SD: 1.5±1.44)

As it is observed laminin expression in hypothyroid group rather than control group increases significantly and in hyperthyroid group no significant difference was observed

**Graph 7:**
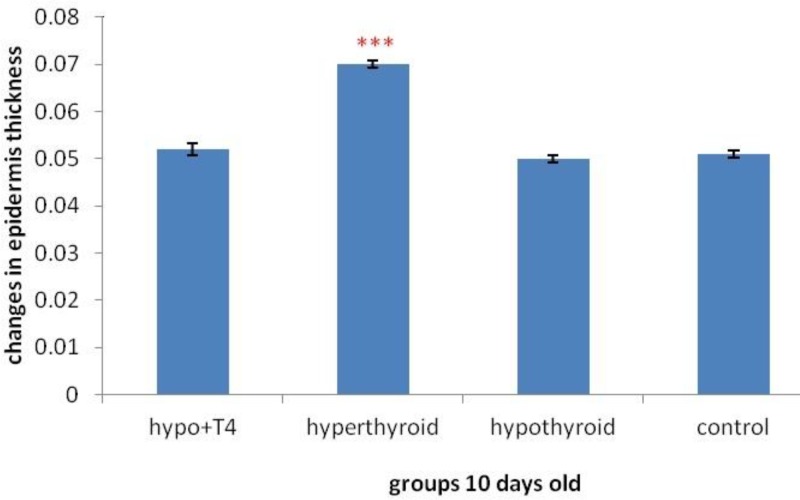
Epidermis thickness changes in 10 day old newborns (***P<0.001), (Mean±SD: 0.06±1.44)

**Graph 8:**
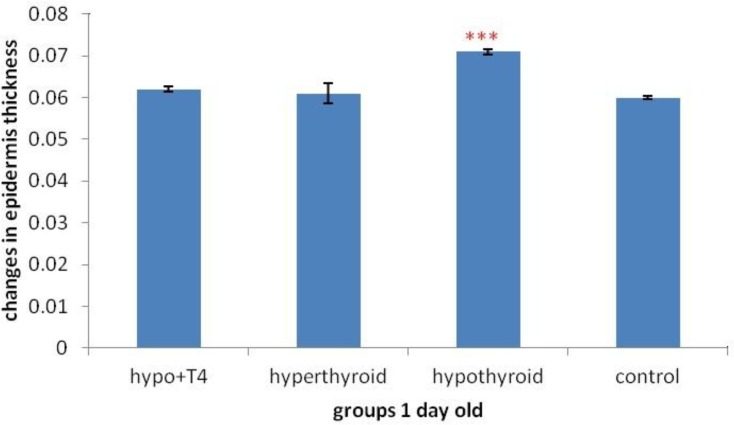
Epidermis thickness changes in 1 day old newborns (***P<0.001), (Mean±SD: 0.65±1.44)

As it is observed in hypothyroid group rather than control group significant increase and in hyperthyroid group no significant difference was observed

**Graph 9:**
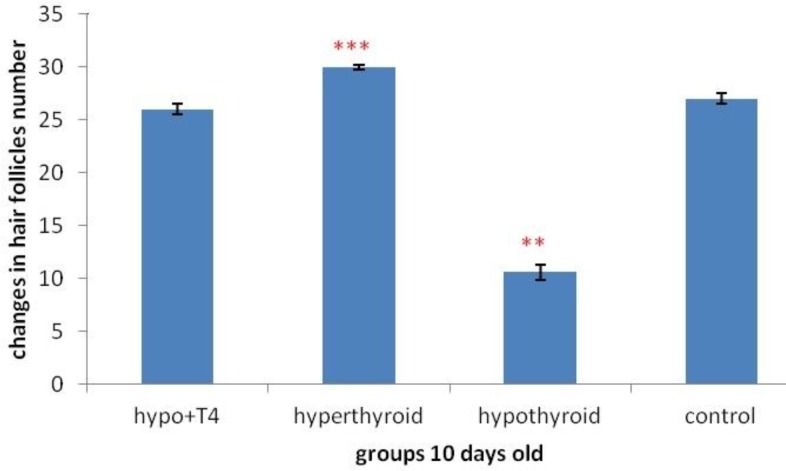
The changes of hair follicle numbers in area 0.044 mm^2^ (***P<0.001 , **P<0.01)

As it is observed hair follicle numbers in hypothyroid group rather than control group decreased and in hyperthyroid group increased significantly

**Graph 10:**
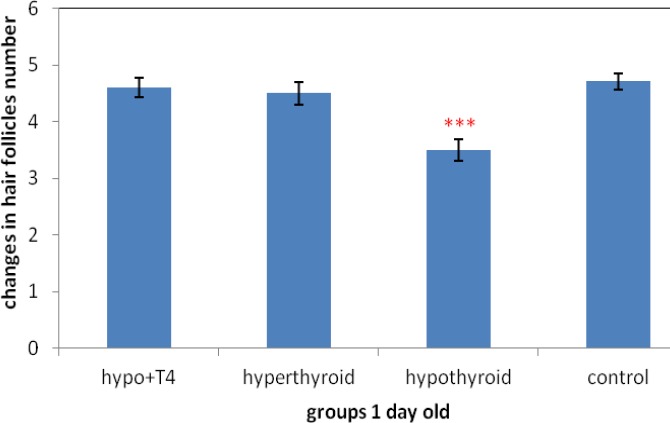
The changes of hair follicle numbers in area of 0.044 mm^2^ (***P<0.001)

As it is observed hair follicle numbers in hypothyroid group rather than control group decreased significantly and in hyperthyroid group no significant difference was observed

Although, in 1 day old newborns, laminin expression increased significantly, in hypothyroid and hyperthyroid groups rather than control group (respectively *P*=0.007 and *P*=0.014) (Figure 2 & Graph 1). But in no significant difference was observed in treated hypothyroid with levothyroxin group in comparison with control group (*P*=1).

Laminin expression was investigated in different layers of epidermis. The results showed that in stratum cornea layer, in 1 day old and 10 day old newborns no significant difference was observed in all mentioned groups (Figure 3). 

But in other layers of epidermis, in 10 day old newborns, in hypothyroid group significant increase was observed rather than control groups (*P*=0.002) and in other groups no significant difference was observed (Figure 3 & Graph 2). Although in 1 day old newborns, laminin expression just in hyperthyroid group in comparison with control group increased significantly (*P*=0.002) and in other groups no significant difference was observed (Figure 4 & Graph 3). 

The investigation of laminin expression in dermis connective tissue showed that in 10 day old newborns, laminin expression increases significantly in hypothyroid group in comparison with control group (*P*=0.007) and in hyperthyroid group in comparison with control group significant decrease was observed (*P*=0.002) (Figure 3 & Graph 4). Also in 1 day old newborns, significant increase was observed laminin expression in hypothyroid group in comparison with control group (*P*=0.002) and significant decrease was observed in hyperthyroid group in comparison with control group (*P*=0.03) (Figure 4 & Graph 4). While in both 1 day old group and 10 day old group no significant difference was observed in treated hypothyroid with levothyroxin in comparison with control group. 

Also Laminin expression was investigated in connective tissue of hypodermis. The results showed, significant increase of laminin expression in hypothyroid groups rather than control group was observed (*P*=0.001) and in hyperthyroid group significant decrease was observed in 10 days old newborns (*P*=0.001) (Figure 5 & Graph 5).

While in 1 day old newborns, laminin expression increases significantly in hypothyroid group in comparison with control group (*P*=0.001) and in other groups no significant differenc was observed (Figure 6 & Graph 6). 

The result of morphometric studies showed that in 10 day old newborns, epidermis thickness increases significantly in hyperthyroid group in comparison with control group, (*P*=0) but in other groups no significant difference was observed (Graph 9). While in 1 day old newborn, in hypothyroid group in comparison withcontrol group significant increase was observed (*P*=0) in other group no significant difference was observed (Graph 8). 

Also the study of hair follicles number in per 0.044 mm^2^ showed significant decrease in 10 days old newborns, in hypothyroid group in comparison control group (*P*=0) . On the other hand in hyperthyroid group significant increase was observed in comparison control group *P*=0) (Graph 9). In 1 day old newborn just in hypothyroid group rather than control group significant decrease was observed (*P*=0) and in other groups no significant difference was observed (Graph 10). 

## Discussion

Previous studies have shown that thyroid hormones affect on growth and development of many organs (10-14). So dysfunction of thyroid gland causes inappropriate growth of these organs. 

Studies showed dysfunction in maternal thyroid hormones in pregnancy and lactation period can affect on development of newborn’s different organs such as skin. On the other hand, the studies showed all isoforms of the thyroid receptor are present in skin and the presence of these receptors has been proven in epidermal keratinocytes, fibroblasts, outer root sheath cells, dermal papilla, fat gland cells, vascular endothelial cells, Schwann cells and smooth muscle cells (15, 16). So widespread presence of these receptors in different parts of skin must have lead in to dramatic effects of thyroid hormones on skin and its appendices. 

Also thyroid hormones are associated with laminin expression regulation that is an extracellular protein matrix. This been a key signal for neurons migration from external granular layer of cerebellum to internal granular layer provided by laminin joined to astrocytes surfaces. In absence of thyroid hormones, appearance time and area distribution time of laminin was impaired and as a result neurons migration impaired. These disorders were observed repeatedly in people with cretinism (17, 18). Considering previous studies and because of the effect of thyroid hormones on many organs and the effect of these hormones on laminin expression, in this study the effect of thyroid hormones on skin and laminin expression was investigated. 

On the other hand, during pregnancy the need for T_4 _hormone increases and because of increased production of human chorionic gonadotropin hormone and its similarity to TSH, thyroid gland hypotrophy and its hormone production increases (18). In addition to that, during pregnancy, two resources provide embryonic thyroid hormones including mother and the embryo. In human beings, embryo in first trimester is dependent on mother for thyroid hormone supply. 

Thyroid hormones transfer from mother to embryo by placental cord that have deiodinase enzyme for transformation T_4_ to T_3_. But in second and third trimester, in addition to mother, the embryo can produce thyroid hormones. In fact the embryo during 10^th^ to 12^th^ of pregnancy gains the ability of thyroid hormones production (19). So any disorder in thyroid gland function in first trimester of pregnancy can influence the embryo. Considering widespread effects of thyroid hormones in various organs development and because skin is the largest and the most important organ in body protection against external damages, in this study the effect of thyroid hormones on skin development was investigated.

In this investigation, laminin expression changes were studied in different areas of skin in rats which were exposed to different dosages of thyroid hormones by immunohistochemistry technique. 

***Laminin expression in hair follicles***

The previous studies showed laminin-10 is necessary for normal development of hair follicles. The dramatic presence of laminin-10 in basement membrane of growing hair follicles and low level of other types of laminin shows the important role of laminin-10 in hair follicle development (20). Researchers showed the skin of mouse Lama5-/- that doesn’t have any laminin-10, have less hair follicles rather than normal mouse in E16.5. On the other hand, thyroid receptor β_1_ is prominent type of thyroid hormone receptor expressed in human hair follicles. Also it has been shown that thyroid hormones have a main role in hair follicle survival and physiological level of free T_3_ increases hair follicles survival *in vivo* considerably (1). 

The study of laminin expression in hair follicles in 1 day old newborns showed laminin expression in hypothyroid group rather than control group increased and in hyperthyroid group decreased. While in 10 day old newborns no significant difference was observed in all groups rather than control group and this difference between 1 day and 10 day old newborns is maby due to the fact that thyroid hormones don’t affect laminin expression in hair follicles during lactation. In fact because thyroid hormones are necessary for hair survival (20, 1) it can be concluded that following thyroid hormones level reduction and for hair follicles survival, laminin expression increases in hypothyroid group and unlike. In fact it seems that presence of thyroid hormones in hair follicles cause reduction of laminin expression, so the reduction of thyroid hormones level in hypothyroid group can be associated with laminin expression increase. Also laminin expression in 1 and 10 days after birth, in treated hypothyroid with levothyroxin groups rather than control group showed no significant difference meaning that thyroxin hormone is a good replacement for curing hypothyroid people and returning them to normal condition. 

***Laminin expression in dermis and epidermis***

 Previous studies in this field showed basement membrane have different roles in dermal-epidermal joins. Its most important function is tightly connectingdermis and epidermis. One of the key components in basement membrane is laminin-5 that is directly connected to collagen type 7 and form anchoring fibril in papillary dermis. In fact the studies showed laminin-5 is the main factor for dermal-epidermal stability (2). 

On the other hand the studies have shown that physiological concentration of thyroid hormones can accelerate epidermis/stratum cornea ontogenesis. In fact hormone T_3_ is excites from epidermal lipids, which is necessary for a normal stratum cornea formation (1). In addition to this thyroid hormones apply a main regulator role on this layer of epidermis by lamellar granules that are necessary for normal stratum cornea formation (21). Also the presence of hormone T_3_ results in an increase of epidermal growth factor receptor number (EGF) in skin that plays a main role in epidermal growth (21, 22). The Graphs and the results of statistical analysis showed no significant difference in laminin expression in stratum cornea in 1 day and 10 day old newborns, in all groups, showing the changes of thyroid hormones level in these groups during lactation and pregnancy doesn’t have any effect on laminin expression in stratum cornea layer of epidermis and in fact this layer isn’t sensitive to changes of thyroid hormones level. 

About laminin expression in all layers of epidermis, it is suggested that integrin receptors of laminin are present in epidermis and some areas of dermis (13). Also other studies have shown that thyroid hormone T_3_ is one of the main regulators of epidermal growth, differentiation and keratin gene expression. This hormone causes keratosis and lipogenesis. In fact epidermal maturation occurs following thyroid hormones distribution and in presence of good level of epidermal growth factor (22). Considering previous studies and laminin presence in epidermis and thyroid hormones effect on it, Graphs and statistical analysis in this study showed that in 1 day old hypothyroid group rather than control group in all layers of epidermis except stratum cornea no significant difference was observed. But in 10 day old newborn, laminin expression in hypothyroid group rather than control group increased significantly. Also laminin expression in these layers in 1 day old newborns in hyperthyroid group rather than control group increase significantly which means thyroid hormones can affect laminin expression in crucial lactation period. 

About the effect of thyroid hormones on dermis, the studies have shown that hypothyroidism can cause water and mucopolysacharides deposition in dermis that causes light reflection change and so makes a yellowish skin (21). Also in Graves syndrome a common type of thyroid autoimmune diseases, hyaluronic acid aggregation increases in dermis and hypodermis that starts from papillary dermis and expends to deep areas of tissue (21, 24). Also the scientists have shown that many thyroid hormone receptors are present in dermis, subepidermis or near to hypodermis showing wide effect of thyroid hormones on this area (24). 

This study showed in both 1 day and 10 day olds, laminin expression increased in dermis connective tissue, in hypothyroid group rather than control group and in hyperthyroid groups decreased significantly that is probably because of negative regulation of laminin expression by thyroid hormones and showing laminin expression during pregnancy and lactation can be impressed by thyroid hormone levels. 

***Laminin expression changes in fat cells and hypodermis connective tissue***

Previous studies have shown that the adipocytes of hypothyroid rats don’t reply to adrenaline by increasing of glycerol release. In other studies, it was shown that phosphodistrase activity rate in adipose tissue determinates by thyroid hormones (25). 

In other studies researcher showed that the number and dimension of fat cell were 86% and 32% less than control group 6 weeks after thyroidectomy In the rats that received thyroid hormones, fat cells hypertrophy was reduced during time and their hyperplasia reduces until two weeks and then becomes zero (26). This study showed that laminin expression in fat cells and hypodermis in 1 day old newborns significant increase was observed just in hypothyroid group rather than control group and in other groups no significant difference was observed. But in 10 day old newborns in hyperthyroid group rather than control group significant decrease and in hypothyroid group significant increase were observed that means positive regulation of laminin expression was done by thyroid hormones in hypodermis and fat cells.

***Morphometric studies***

About epidermis thickness changes with thyroid hormones level changes, previous studies showed local T_3_ hormone can cause thicker epidermis and more hair follicles in comparison with intraperitoneally T_3_ hormone (27). Another study showed increase in thyroid hormone level causes significant decrease in epidermis thickness. The results showed there is a significant positive relation between blood triiodothyronine and epidermis thickness (28). Another study showed epidermal thickness decreases in hypothyroidism significantly. It was also expressed that epidermal proliferation rate and anabolic activities in epidermis increases in thyrotoxicosis significantly. Another finding was that hyperthyroidism is important more than hypothyroidism in epidermis changes, (29). The result of this study in 10 day old newborns is consistent with Holt PJ and etal work that showed the increase of thyroid hormones level causes epidermis thickness increase and cell proliferation but in hypothyroid group in spite of Holt PJ work, no significant decrease was observed. In 1 day old newborns the results was consist with another study that showed hypothyroidism causes increase in epidermal thickness, That means the changes of thyroid hormone level in lactation and pregnancy period can result in different effects on epidermal thickness. 

About hair follicle number, the previous studies showed hypothyroidism leads to decrease hair growth and hair follicle survival. In fact hypothyroidism causes hair fall and hair number decrease (24, 27). This study showed hyperthyroidism accompany with hair growth and hair survival increase. On the other hand in 10 day old newborns hair follicles number in certain area in hypothyroid group increased and in hyperthyroid group decreased significantly. In 1 day old newborns the results was the same but in hyperthyroid group 10 day old newborns, no significant difference was observed. 

## Conclusion

Because hypothyroidism causes laminin expression increase and hyperthyroidism causes laminin expression decrease, it is concluded that thyroid hormones cause negative regulation of laminin expression which means thyroid hormones level increase causes laminin expression decrease and unlike. So laminin lack probably is a defect and hypothyroidism causes laminin expression increase that is positive, on the other hand the difference in laminin expression in 1 day and 10 day olds in various areas of skin can show different effect of thyroid hormones during lactation and pregnancy.
